# Inflammation in Cardiomyopathies: Cellular Mechanisms Across Cardiac Phenotype

**DOI:** 10.3390/cells15121131

**Published:** 2026-06-22

**Authors:** Antonio Lattanzio, Giulia Marchionni, Giulia Pecci, Federico Ciccarelli, Silvia Stavagna, Jacopo Costantino, Federico Ballatore, Maria Alfarano, Francesco Ciciarello, Cristina Chimenti

**Affiliations:** 1 Department of Medical and Cardiovascular Sciences, Sapienza University of Rome, Viale del Policlinico 155, 00161 Rome, Italy; antonio.lattanzio@uniroma1.it (A.L.); giulia.marchionni.1@studenti.unipd.it (G.M.); pecci.1747716@studenti.uniroma1.it (G.P.); federico.ciccarelli@uniroma1.it (F.C.); silvia.stavagna@uniroma1.it (S.S.); jacopo.costantino@uniroma1.it (J.C.); federico.ballatore@uniroma1.it (F.B.); m.alfarano@policlinicoumberto1.it (M.A.); francesco.ciciarello@uniroma1.it (F.C.); 2 Department of Medicine, University of Padua, 35128 Padova, Italy; 3 Department of Cardiology, San Pietro Fatebenefratelli Hospital, 00189 Rome, Italy

**Keywords:** cardiomyopathies, myocardial inflammation, myocardial fibrosis, innate immunity, hypertrophic cardiomyopathy, Anderson–Fabry disease, cardiac amyloidosis, arrhythmogenic cardiomyopathy, dilated cardiomyopathy

## Abstract

Cardiomyopathies are traditionally classified by structural and genetic phenotypes, but emerging evidence highlights chronic myocardial inflammation as a pivotal driver of disease progression across different etiologies. This review synthesizes the current literature on the cellular and molecular inflammatory mechanisms underlying hypertrophic cardiomyopathy, Anderson–Fabry disease, cardiac amyloidosis, arrhythmogenic cardiomyopathy, and dilated cardiomyopathy. Across these distinct conditions, endogenous triggers such as metabolic substrates, misfolded amyloid fibrils, mechanical stress, or viral genomes act as damage-associated molecular patterns. These stimuli activate innate and adaptive immune cascades, notably the Toll-like receptors, the NF-κB pathway, and the NLRP3 inflammasome. This immune activation establishes a pro-inflammatory microenvironment that promotes fibroblast reprogramming, myocardial edema, and progressive fibrotic or fibro-fatty remodeling. Inflammation is an active, core pathophysiological mechanism rather than a passive secondary bystander in cardiomyopathies. Recognizing these shared immune pathways provides a framework for improved risk stratification and highlights the potential for targeted immunomodulatory therapies to alter disease trajectories.

## 1. Introduction

Cardiomyopathies encompass a heterogeneous group of myocardial disorders traditionally classified by distinct morphological and functional phenotypes, such as hypertrophic, dilated, and arrhythmogenic patterns. Historically, the pathogenesis of these conditions has been primarily attributed to genetic mutations affecting sarcomeric and desmosomal proteins, or to the passive accumulation of metabolic substrates and misfolded proteins. Consequently, clinical models and therapeutic strategies have largely focused on the structural consequences and on alleviating of mechanical stress.

However, a paradigm shift is currently underway in cardiovascular medicine. Accumulating clinical, histological, and molecular evidence indicates that chronic, low-grade myocardial inflammation is not merely a secondary bystander, but an active driver of disease progression and adverse remodeling. Whether triggered by several mechanisms often coexisting, such as metabolic storage, fibril deposition, mechanical stress, or viral persistence, myocardial injury consistently activates innate and adaptive immune cascades. Damage-associated molecular patterns (DAMPs) engage pattern-recognition receptors, such as Toll-like receptors, thereby activating core signaling pathways, including NF-κB and the NLRP3 inflammasome. This immune activation fosters a deleterious autocrine–paracrine loop of cytokine release, oxidative stress, and fibroblast reprogramming, ultimately culminating in maladaptive tissue remodeling, progressive fibrosis, and functional decline.

This review aims to comprehensively synthesize the current state of research regarding the cellular mechanisms of inflammation across major cardiac phenotypes. By highlighting the shared and distinct inflammatory pathways in hypertrophic cardiomyopathy, Anderson–Fabry disease, cardiac amyloidosis, arrhythmogenic right ventricular cardiomyopathy, and dilated cardiomyopathy, we underscore the crucial role of the immune system in disease progression and evaluate the potential of immunomodulatory strategies as promising novel therapeutic interventions.

Restrictive cardiomyopathy and non-dilated left ventricular cardiomyopathy were not included, as current evidence does not consistently support a primary mechanistic role of inflammation in shaping their cardiac phenotype, but rather suggests a secondary or end-stage involvement.

## 2. Hypertrophic Cardiomyopathy

Hypertrophic cardiomyopathy (HCM) is a common inherited cardiac disease (prevalence ~1:200–500) characterized by sarcomere dysfunction leading to excessive pathological hypercontractility and impaired diastolic relaxation, which phenotypically result in left ventricular (LV) hypertrophy, diastolic dysfunction, and myocardial fibrosis [1,2]. Its genetic background predominantly involves mutations in sarcomeric proteins, most commonly β-myosin heavy chain and myosin-binding protein C; however, in recent years, non-sarcomeric variants have also been associated with this clinical phenotype [3].

Traditionally, HCM has been regarded primarily as a genetic disorder of the cardiac muscle; however, accumulating evidence suggests that inflammation plays a significant modulatory role in disease pathophysiology and progression. This hypothesis is supported by the identification of a chronic low-grade inflammatory state in HCM, demonstrated by histological analyses, circulating inflammatory mediators, and proteomic studies [4,5].

Compared with healthy individuals, patients with HCM exhibit higher circulating levels of inflammatory cytokines and acute-phase reactants. In a study by Fang et al., 17 cytokines and chemokines were measured in 50 HCM patients, revealing significantly increased plasma levels of TNF-α and IL-6 compared with controls. In the same study, IL-6 showed a positive correlation with the extent of regional myocardial fibrosis assessed by cardiac MRI, whereas MCP-1 (and stromal cell-derived factor-1) correlated with the degree of diffuse fibrosis. Several inflammatory markers, including TNF-α, IL-6, serum amyloid P, and IL-10, were also associated with indices of diastolic dysfunction [6].

In a surgical pathology series of septal myectomy specimens from 204 HCM patients, up to 48% of samples displayed foci of mild chronic inflammatory infiltrates within the myocardium [7]. Kuusisto et al. further demonstrated variable degrees of myocyte hypertrophy and disarray accompanied by inflammatory cell infiltration and activation of the NF-κB signalling pathway. In these patients, circulating hs-CRP and several interleukins (IL-1β, IL-6, IL-1RA, IL-10) were significantly elevated compared with control subjects. Importantly, the extent of myocardial inflammatory infiltrates was positively correlated with the degree of myocardial fibrosis, as assessed both histologically and by cardiac MRI [8].

Similarly, a recent study including 102 HCM patients who underwent septal myectomy found that higher levels of IL-2 and TNF-α were associated with greater myocardial fibrosis on histological analysis [8]. Moreover, elevated serum hs-CRP levels were associated with greater fibrotic burden in myocardial tissue samples. Collectively, these findings suggest that fibrosis in HCM is not merely a passive consequence of myocyte hypertrophy but rather an active process closely intertwined with inflammation.

Notably, increased levels of pro-inflammatory and pro-apoptotic mediators have been reported in patients who progress to the end-stage dilated phenotype [9]. An endomyocardial biopsy study by Frustaci A. et al. showed the presence of myocarditis in 67% of patients with acute clinical deterioration in half of cases related to a myocardial viral infection [10].

These observations in humans are paralleled by findings in animal models, demonstrating that the transition from hypertrophy to a dilated end-stage phenotype is accompanied by upregulation of pro-inflammatory and pro-fibrotic genes [11].

The proposed pathophysiological model linking HCM progression to inflammation involves an initial insult driven by mechanical stress, disorganized sarcomeric architecture, and myocardial ischemia, which induces upregulation of the NF-κB pathway. This, in turn, activates inflammatory cascades and cytokine release, leading to fibroblast activation and myocardial fibrosis.

In a 10-year longitudinal study, Pelliccia et al. measured NF-κB activity in peripheral blood mononuclear cells of patients with asymptomatic or mildly symptomatic HCM and found that higher baseline NF-κB levels were associated with a significantly increased risk of heart failure progression over the following decade [12]. Similarly, upregulation of the Ras–MAPK pathway identified by proteomic analyses, as well as elevated neutrophil-to-lymphocyte ratios, were able to discriminate HCM patients from controls and were significantly correlated with symptom burden and disease severity [13,14].

Overall, these findings suggest that a heightened inflammatory state portends a worse prognosis in HCM. Although further studies are required to validate specific inflammatory biomarkers, the available evidence indicates that inflammation may influence the likelihood of developing heart failure symptoms or experiencing sudden arrhythmic events. Indeed, CMR-based studies have demonstrated that increased T2-weighted signal intensity in HCM is associated with a higher arrhythmic risk [15,16,17].

**Figure 1:**
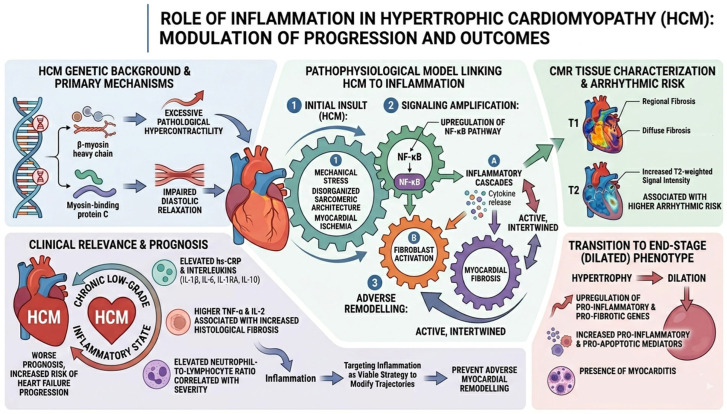
Conceptual framework detailing the role of inflammation in the pathogenesis, progression, and clinical outcomes of Hypertrophic Cardiomyopathy (HCM). The schematic illustrates the complex interplay among genetic predisposition, local inflammatory signaling, and structural cardiac remodeling. **Left panels: Genetic background and clinical relevance:** Primary pathogenic mutations (e.g., affecting β-myosin heavy chain and myosin-binding protein C) induce pathological hypercontractility and impaired diastolic relaxation. This altered biomechanics promotes a chronic, low-grade systemic inflammatory state. Elevated circulating biomarkers—including hs-CRP, various interleukins (IL-1β, IL-6, IL-10, IL-1RA), TNF-α, and an increased neutrophil-to-lymphocyte ratio—strongly correlate with histological fibrosis, disease severity, and a worse prognosis regarding heart failure progression. **Central panel: pathophysiological mechanisms:** The initial mechanical stress and ischemia inherent to HCM trigger downstream signaling amplification, notably through the upregulation of the NF-κB pathway. This initiates an active, intertwined cascade of cytokine release and fibroblast activation, creating a vicious cycle that accelerates myocardial fibrosis and adverse ventricular remodeling. **Right panels, imaging and phenotypic transition**: Tissue characterization via Cardiovascular Magnetic Resonance (CMR) reflects these microstructural changes; T1 mapping identifies regional and diffuse fibrosis, while increased T2-weighted signal intensity indicates edema/active inflammation, both of which are associated with heightened arrhythmic risk. Ultimately, sustained upregulation of pro-inflammatory and pro-apoptotic mediators can drive the transition from a hypertrophic to a high-risk, end-stage dilated phenotype. Consequently, targeted anti-inflammatory therapies represent a viable future strategy to prevent adverse myocardial remodeling and modify disease trajectories.

Ongoing research is therefore needed to clarify whether targeting inflammation may represent a viable strategy to modify disease trajectories; if this hypothesis is confirmed, immune-modulating therapies could emerge as novel interventions to prevent adverse myocardial remodelling in HCM [*Figure 1*].

## 3. Anderson–Fabry Disease

Anderson–Fabry disease (FD) is a rare X-linked lysosomal storage disorder caused by mutations in the GLA gene, resulting in deficiency in the enzyme α-galactosidase A and systemic accumulation of glycosphingolipids, primarily globotriaosylceramide (Gb3) and its deacylated form globotriaosylsphingosine (lyso-Gb3) [18]. Accumulation occurs in virtually all cardiac cell types, including cardiomyocytes, endothelial cells, vascular smooth muscle cells, fibroblasts, and the conduction system, leading to structural and functional abnormalities such as left ventricular hypertrophy, myocardial fibrosis, diastolic dysfunction, arrhythmias, and progressive heart failure [19].

Clinically, FD presents along a spectrum. Classic FD, with near-absent enzyme activity, manifests in childhood with systemic symptoms including acroparesthesias, angiokeratomas, and hypohidrosis. In late-onset variants, residual enzyme activity often leads to predominantly cardiac, renal, or cerebrovascular manifestations later in life. Cardiac involvement remains the leading cause of morbidity and mortality in adults with FD [20].

While lysosomal storage was historically considered the principal driver of cardiac injury, emerging evidence highlights a more complex pathogenesis. Substrate accumulation triggers secondary mechanisms, including oxidative stress, mitochondrial dysfunction, endothelial impairment, and chronic inflammation, which collectively contribute to myocardial remodelling and dysfunction [21].

In FD, inflammation is typically chronic and low-grade, linked to innate immune activation. Gb3 and lyso-Gb3 may act as damage-associated molecular patterns (DAMPs), engaging pattern-recognition receptors such as Toll-like receptor 4 and promoting cytokine release (IL-1β, IL-6, TNF-α) and oxidative stress. These inflammatory signals have been observed in peripheral immune cells from FD patients and are increasingly recognized as contributors to cardiac injury and fibrotic remodelling [22].

Advanced cardiac imaging suggests that inflammation may precede or accompany structural changes in FD, detectable even before overt hypertrophy or late gadolinium enhancement (LGE) on cardiac magnetic resonance imaging (CMR), indicating an early role in disease progression [23].

Fabry cardiomyopathy is no longer considered a passive consequence of substrate accumulation. Instead, glycosphingolipid storage initiates a cascade of secondary processes, including endothelial dysfunction, oxidative stress, myocardial remodelling, and chronic inflammation [24,25]. Lyso-Gb3 is biologically active, promoting pro-inflammatory and pro-hypertrophic signalling in cardiomyocytes, endothelial cells, and vascular smooth muscle cells, thereby amplifying myocardial stress and remodelling [25,26].

Clinically, Fabry cardiomyopathy involves chronic activation of innate immune pathways. Accumulated substrates act as endogenous danger signals, sustaining cytokine production and low-grade inflammation, which contributes to hypertrophy, microvascular dysfunction, and progression toward fibrosis [19,27].

Histological evidence supports this model: endomyocardial biopsies demonstrate immune cell infiltration consistent with myocarditis in a substantial subset of patients, particularly those with advanced cardiac involvement. These findings correlate with disease severity and adverse outcomes [28]. Non-invasive imaging, including CMR and positron emission tomography (PET), reveals myocardial edema and metabolic activity indicative of inflammation, which may precede fibrosis or systolic dysfunction [24,29]. Together, these observations suggest a self-perpetuating inflammatory process linking metabolic storage to clinical progression.

Inflammation in FD involves multiple cardiac compartments and displays heterogeneous regional distribution. Cardiomyocytes actively contribute to the inflammatory milieu: lysosomal storage induces stress responses that trigger pro-inflammatory signalling and hypertrophy, even in the absence of pressure overload [25,26].

Endothelial cells and the coronary microvasculature are particularly affected. Gb3 accumulation promotes a pro-inflammatory and pro-thrombotic phenotype, impairing microvascular perfusion and leading to angina-like symptoms, exercise intolerance, and ischemia without epicardial coronary obstruction [24,29]. Chronic microvascular inflammation may precede localized myocardial injury.

Immune cell infiltration, including T lymphocytes and macrophages, is observed in a subset of patients, often accompanied by persistently elevated troponin and progressive fibrosis. Unlike acute viral myocarditis, this inflammation is chronic and low-grade, consistent with the concept of Fabry cardiomyopathy as a chronic inflammatory cardiomyopathy [28,30].

Importantly, inflammation is regionally selective, frequently involving the inferolateral left ventricular wall. This pattern mirrors LGE on CMR and reflects the interaction of microvascular dysfunction, localized inflammation, and mechanical stress. Over time, inflamed regions evolve into replacement fibrosis, strongly associated with arrhythmias and adverse outcomes [24,29].

Inflammation in FD has clear clinical relevance, as reflected by imaging findings, circulating biomarkers, and outcomes. CMR allows assessment of storage (T1 mapping), inflammation (T2 mapping), and fibrosis (LGE), while PET can detect metabolic activity consistent with active inflammation [24,29,31].

Persistent elevation in high-sensitivity troponin is common and correlates with myocardial inflammation and fibrosis rather than acute ischemia. Additional markers such as IL-6 and CRP further support the presence of chronic inflammation, which parallels imaging abnormalities and clinical deterioration [24,25,30].

Regions of inflammation and subsequent fibrosis are associated with ventricular arrhythmias, progressive heart failure, and increased mortality. The extent and localization of LGE are robust predictors of adverse outcomes and aid risk stratification [29].

**Figure 2:**
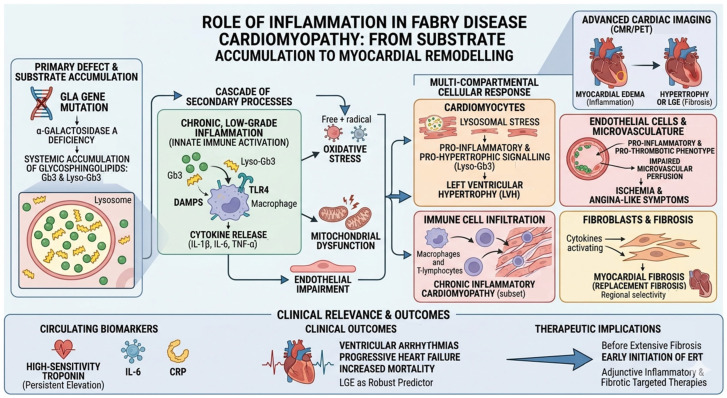
**Pathophysiological cascade of inflammation-driven myocardial remodeling in Fabry Disease Cardiomyopathy.** The schematic illustrates the progression from primary genetic defect to multi-compartmental cardiac dysfunction. **Left panel, primary defect and substrate accumulation**: A mutation in the *GLA* gene leads to alpha-galactosidase A deficiency, causing the systemic and lysosomal accumulation of glycosphingolipids, predominantly globotriaosylceramide (Gb3) and globotriaosylsphingosine (lyso-Gb3). **Central panel: secondary Signaling Cascades**: These accumulated sphingolipids act as damage-associated molecular patterns (DAMPs), binding to Toll-like receptor 4 (TLR4) on macrophages. This triggers innate immune activation and a chronic, low-grade inflammatory state characterized by the continuous release of pro-inflammatory cytokines (IL-6, TNF-alpha). Parallel secondary processes include elevated oxidative stress (free radical production) and profound mitochondrial dysfunction, contributing to early endothelial impairment. **Right panels: Multi-compartmental Cellular Response & Imaging:** The inflammatory milieu drives distinct responses across cardiac tissue compartments: (1) cardiomyocytes experience lysosomal stress and pro-hypertrophic signaling, resulting in left ventricular hypertrophy (LVH); (2) endothelial cells adopt a pro-thrombotic phenotype, impairing microvascular perfusion and causing angina-like symptoms; and (3) active immune cell infiltration stimulates fibroblast activation, leading to regionally selective replacement myocardial fibrosis. Advanced cardiac imaging (CMR/PET) maps this temporal evolution from early myocardial edema to established fibrosis (LGE). **Bottom panel: Clinical Relevance:** The progression of this inflammatory cardiomyopathy is reflected by circulating biomarkers (persistently elevated hs-Troponin, IL-6, CRP) and culminates in severe clinical outcomes, including ventricular arrhythmias and progressive heart failure. Therapeutic implications emphasize the critical need for early initiation of Enzyme Replacement Therapy (ERT) prior to the onset of extensive fibrosis, alongside the potential integration of adjunctive therapies explicitly targeting the inflammatory and fibrotic pathways.

Therapeutically, enzyme replacement therapy is most effective when initiated before extensive fibrosis, suggesting that early inflammatory activity represents a clinically relevant window for intervention [24,25]. Recognition of inflammation as a central pathogenic mechanism underscores the potential for adjunctive therapies targeting inflammatory and fibrotic pathways *[Figure 2]*.

## 4. Cardiac Amyloidosis

Cardiac amyloidosis (CA) encompasses a group of infiltrative cardiomyopathies characterized by the deposition of misfolded protein fibrils—most commonly immunoglobulin light chain (AL) or transthyretin (aTTR)—within the cardiac interstitium. Historically, the disorder has been conceptualized primarily as a problem of mechanical stiffening: progressive amyloid accumulation increases myocardial wall thickness, disrupts normal chamber compliance and culminates in restrictive physiology and heart failure [18].

However, major advances in molecular biology, immunology and functional cardiac imaging challenge this purely structural paradigm. Increasingly, CA is understood to involve not only the passive deposition of insoluble fibrils but also an active, dynamic inflammatory process. In AL amyloidosis, soluble free light chains exert direct cardiotoxicity independent of fibril burden, initiating inflammatory and oxidative injury that accelerates ventricular dysfunction [32]. In aTTR disease—including both wild-type (aTTRwt) and hereditary (aTTRv) forms—recent evidence indicates that mutant and wild-type TTR aggregates activate innate immune pathways and reprogram fibroblasts [33,34].

Clinically, myocardial edema detected by T2 mapping predicts prognosis in CA [35], while tissue biopsy findings of inflammatory infiltrates independently predict mortality in AL disease [34]. Additionally, circulating markers such as IL-6 correlate with disease severity in aTTR [33]. Together, these data highlight inflammation as a mechanistic hallmark and potential therapeutic target across amyloid types.

### 4.1. AL Cardiac Amyloidosis

In AL amyloidosis, monoclonal plasma cells produce structurally unstable immunoglobulin light chains that circulate systemically and deposit as amyloid fibrils. Unlike aTTR, where fibril toxicity predominates, AL disease features an additional pathogenic component: soluble light chains are intrinsically cardiotoxic.

Seminal in vitro work demonstrates that amyloidogenic light chains activate oxidative stress pathways, mitochondrial depolarization, unfolded protein response signalling, and pro-inflammatory transcriptional reprogramming. Jordan et al.’s study [32] showed that pathogenic AL light chains induce robust NF-kB-dependent cytokine production, including IL-6 and TNF-alpha, in human cardiomyocytes and mesenchymal stromal cells. This suggests an autocrine–paracrine amplification loop of inflammation and injury.

Early-stage aggregates—including soluble oligomers—behave as damage-associated molecular patterns (DAMPs) capable of activating, such as Toll-like receptors (TLR2, TLR4) and NLP3 inflammasome activation results in caspase-1 activity and maturation of IL-1beta and IL-18, linking AL amyloidosis to other sterile inflammatory amyloid diseases.

Siegismund et al. [35] demonstrated that inflammatory infiltrates—macrophages, lymphocytes, activated stromal cells—are common in AL cardiomyopathy and predict mortality independently of amyloid burden. This was among the first studies to show that inflammation directly contributes to prognosis.

AL amyloidosis frequently coexists with plasma cell dyscrasias, including multiple myeloma. These disorders exhibit a chronic pro-inflammatory state characterized by elevated IL-6 and other cytokines that promote plasma cell proliferation and may exacerbate cardiac inflammation [36].

Modern models conceptualize AL cardiac involvement as the convergence of soluble light-chain toxicity (inflammatory, oxidative and metabolic injury) and amyloid fibril deposition (mechanical stiffening and impaired diastolic function). Inflammation bridges these processes, explaining why rapid hematologic response often produces dramatic clinical improvement: inflammatory injury is reversible if treated early.

### 4.2. ATTR Cardiac Amyloidosis

ATTR amyloidosis includes both wild-type (aTTRwt) and variant (aTTRv) forms and was long considered a degenerative, non-inflammatory protein deposition disorder. However, accumulating evidence demonstrates that aTTR amyloidosis involves systemic and myocardial inflammatory activation contributing to disease progression.

In aTTRv, genetic variants destabilize the TTR tetramer, promoting misfolding and aggregation. Mutant oligomeric TTR species interact with immune pattern-recognition receptors.

Isoguchi et al. [33] demonstrated that patients with familiar aTTR show systemic inflammatory activation, including elevated cytokines and mutated TTR can directly activate macrophages and dendritic cells. These findings support a model in which TTR oligomers function as damage-associated molecular patterns (DAMPs) initiating innate immune responses.

Even in aTTRwt, TTR fibrils provoke monocyte/macrophage activation, endothelial stress and oxidative injury. Dittloff et al. [37] further showed that TTR fibrils trigger cytoskeletal disruption in cardiac fibroblasts, upregulation of inflammatory genes and pro-fibrotic signalling. This indicates that inflammation is intrinsic to TTR amyloid biology, not merely a consequence of hereditary variants.

In response to TTR fibrils, fibroblasts undergo transcriptional reprogramming, characterized by myofibroblast transformation, increased ECM production, secretion of inflammatory mediators. These alterations promote progressive myocardial stiffening and chronic inflammation.

Although less directly toxic than AL light chains, aTTR fibrils disrupt mitochondrial dynamics, sarcomeric integrity and calcium handling. These stress responses can intensify inflammatory signalling and contribute to functional decline.

Hein et al. [34] reported that elevated IL-6 levels in aTTR cardiomyopathy correlate with worse functional class and reduced survival. This positions IL-6 as a marker of disease severity and suggests a systemic inflammatory phenotype in a subset of patients. As in AL disease, aTTR patients show myocardial edema detectable on T2 mapping [38], reflecting active inflammatory injury. Thus, aTTR may progress through sequential phases: tetramer destabilization, immune activation, amyloid fibril deposition and fibrosis and diastolic dysfunction [39].

### 4.3. Shared Inflammatory Pathways in AL and aTTR Amyloidosis

**Figure 3:**
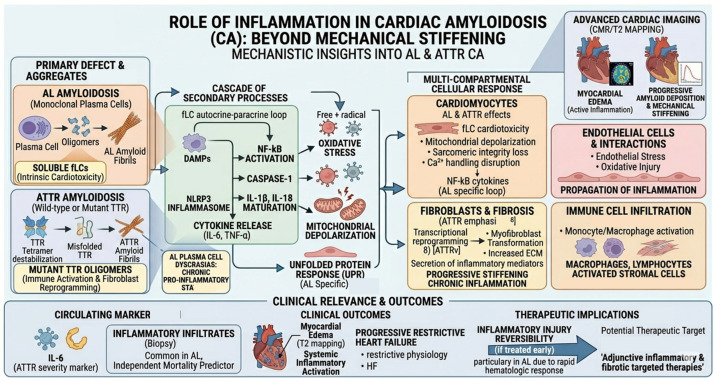
**Mechanistic paradigm of inflammation in Cardiac Amyloidosis (CA), elucidating the pathways beyond mechanical stiffening in Light Chain (AL) and Transthyretin (aTTR) subtypes.** The schematic highlights the transition from proteotoxicity to systemic and local inflammatory cascades. **Left panels: Primary Defects and Proteotoxicity:** The pathogenesis involves distinct precursor proteins: in AL amyloidosis, monoclonal plasma cells produce amyloidogenic free light chains (fLCs) which form oligomers and fibrils, with soluble fLCs exerting direct, intrinsic cardiotoxicity. In aTTR amyloidosis, the destabilization of wild-type or mutant transthyretin (TTR) tetramers yields misfolded oligomers that specifically drive immune activation and fibroblast reprogramming. **Central panel: Secondary Signaling and Inflammatory Cascades:** Early-stage aggregates from both subtypes act as damage-associated molecular patterns (DAMPs). They trigger the NLRP3 inflammasome and activate the NF-κB pathway, leading to Caspase-1 activation and the maturation and release of pro-inflammatory cytokines (e.g., IL-1β, IL-18, IL-6, TNF-α). This molecular cascade promotes severe oxidative stress (free radical generation) and mitochondrial depolarization, while specifically triggering the Unfolded Protein Response (UPR) in the AL subtype. **Right panels: Multi-compartmental Cellular Responses and Imaging:** This toxic and inflammatory milieu drives a multifaceted cellular response: (1) cardiomyocytes experience mitochondrial depolarization, loss of sarcomeric integrity, and disruption of Ca2+ handling; (2) fibroblasts undergo transcriptional reprogramming (with an emphasis in aTTR) into myofibroblasts, increasing extracellular matrix (ECM) production and progressive tissue stiffening; and (3) endothelial stress fosters microvascular injury. A shared feature is the infiltration of activated monocytes/macrophages and lymphocytes, which propagates local inflammation. Advanced cardiac imaging (such as CMR with T2 mapping) captures the resulting myocardial edema—an indicator of active inflammation preceding dense mechanical stiffening. **Bottom panel: Clinical Relevance and Therapeutic Horizons:** Circulating markers (e.g., IL-6 as a severity marker in aTTR) and biopsy-proven inflammatory infiltrates are strongly associated with progressive restrictive heart failure and serve as independent predictors of mortality. The potential reversibility of the early inflammatory injury—particularly in AL CA following rapid hematologic response—underscores the emerging rationale for early intervention with adjunctive inflammatory and fibrotic targeted therapies.

Despite distinct etiologies, AL and aTTR amyloidosis share convergent inflammatory mechanisms influencing cardiac injury *[Figure 3]*.

Amyloid fibrils and oligomers activate Toll-like receptors (TLR2, TLR4) and induce NF-kB-mediated cytokine release; NLRP3 inflammasome causes IL-1beta and IL-18 maturation. The result is a chronic sterile inflammatory state similar to other protein misfolding diseases.

## 5. Arrhythmogenic Right Ventricular Cardiomyopathy (ARVC)

Arrhythmogenic cardiomyopathy (ACM) is an inherited myocardial disease characterized by the progressive fibro-fatty replacement of cardiac tissue, historically affecting the right ventricle but frequently involving the left ventricle or presenting with biventricular disease [40]. Myocardial tissue alterations start from the epicardium and extend toward the endocardium to become transmural, resulting in progressive wall thinning; myocardial scarring predisposes to impairment of ventricular systolic function and increased risk of ventricular arrhythmia and sudden cardiac death [41]. Therefore, ACM is associated with poor prognosis and high heart transplant rates, especially in young patients and athletes.

Numerous studies have identified the genes most frequently implicated in the ACM pathogenesis; in genotype-positive patients, mutations involve desmosomal genes such as plakophilin-2 (PKP2), desmoplakin (DSP), junctional plakoglobin (JUP), desmoglein-2 (DSG2), and desmocollin-2 (DSC2) [42]. Pathogenic variants in desmosomal genes impair cell–cell adhesion, leading to cardiomyocyte detachment and subsequent fibro-adipose replacement. Although mutations in desmosomal genes represent by far the most prevalent genetic variants associated with ACM, pathogenic variants in non-desmosomal genes have also been identified in genes encoding proteins involved in cytoskeletal organization, calcium handling, sodium ion transport, and cytokine signaling; notable non-desmosomal genes implicated in ACM include DES (encoding desmin), LMNA (encoding lamin A/C), TMEM43 (Encoding transmembrane protein-43), TTNv (encoding titin), and FLNC (encoding filamin C) [42].

Although it has traditionally been considered a structural disorder related to genetic defects, growing evidence suggests that inflammation plays a relevant role in its pathogenesis and progression. Nevertheless, it remains unclear whether inflammation plays a primary role or is secondary to myocyte necrosis, thereby contributing to disease progression. Since the 1990s, the presence of inflammatory infiltrates at the myocardial level has been recognized in autopsy analyses of patients affected by ACM. Basso et al. [43] demonstrated that scattered foci of lymphocytes associated with myocardial cell death were observed in 20 cases in their series (67%); however, whether inflammation represents a primary event or a reaction to cardiomyocyte necrosis is unknown. Following the identification of defects in desmosomal components as the genetic basis of the disease, it was hypothesized that the genetic mutations could predispose to myocyte detachment and death, and that significant myocyte loss could trigger an inflammatory response [43]. Myocardial damage activates both innate and adaptive immune responses by recruiting macrophages, mast cells, B cells, and T cells, with monocyte-derived macrophages infiltrating and proliferating in the damaged tissue. These macrophages interact with cardiomyocytes, fibroblasts, and endothelial cells via pro-inflammatory mediators such as TNF-α, TGF-β, interleukins, and matrix metalloproteinases, propagating inflammation and stimulating cardiac remodelling and fibrosis. Inflammatory pathways, including TLR, NFκB, MAPK, and caspase-1 inflammasomes, contribute to oxidative stress and cytokine release. Macrophages also directly participate in collagen deposition and electrical conduction through CX43-mediated coupling with cardiomyocytes. Overall, these findings highlight that inflammation has an active role in the progression of remodelling, suggesting that targeting immune pathways may offer novel therapeutic strategies [43,44].

More recently, Campuzano et al. [45] demonstrated an association between the extent of inflammation in the ventricular myocardium of ACM samples and structural cardiac abnormalities, as reflected in the severity of fibro-fatty replacement and prevalence of biventricular involvement, indicating that the inflammatory process may act as a modulator of disease severity in ARVC. Nevertheless, the causal and prognostic roles of inflammation have yet to be proved.

Recent studies have demonstrated that the impact of inflammation and autoimmunity differs according to the casual variant gene, with most data derived from desmosomal forms; non-desmosomal subtypes require further study. Inflammatory processes appear to be more prevalent in left-dominant ACM and gene-dependent, with the DSP-cardiomyopathy showing the highest degree of inflammation [46].

It has also been hypothesized that viral infections can determine the progression of the disease due to inflammatory response. Some case reports have detected different cardiotropic viruses in samples of patients with ACM. Bowles et al. report in a series of 12 patients that cardiotropic viruses were more frequently detected in ARVC patients than in control subjects. However, the role of these viruses in inducing inflammation and ACM pathogenesis remains unclear [47].

In 2007, the term “hot phase” was used for the first time by Sen-Chowdhry et al. to describe periodic exacerbations, sometimes clinically evident, of a gradual and continuous process in an otherwise quiescent disease. Since then, several studies have reported that inflammatory cells are particularly abundant in the affected myocardial tissue during the so-called hot phases of ACM [48]. A 2022 review by Bariani et al. [49] reported that patients experiencing hot phase episodes were predominantly young (mean age 26 ± 14 years), with a significant representation of pediatric cases. Exercise acts as a trigger in 50% of cases. The review also showed that DSP is the gene most frequently associated with the presentation through hot phases, while PKP2 and DSG2 variants were identified in 17% of cases. These findings underscore the importance of a timely differential diagnosis between myocarditis and hot phases, only possible through an EMB, to achieve an accurate diagnosis and how the overlap between these two conditions is related to the role of inflammation in their pathogenesis.

**Figure 4:**
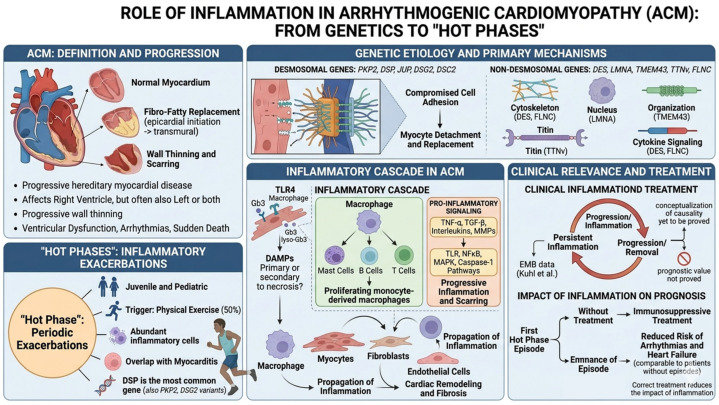
**Comprehensive overview of the pathophysiological mechanisms and the role of inflammation in Arrhythmogenic Cardiomyopathy (ACM), highlighting the transition from genetic etiology to acute inflammatory “hot phases.”** The schematic maps the disease trajectory from primary molecular defects to cyclical inflammatory exacerbations and fibrotic remodeling. **Top panels: Definition and Genetic Etiology:** ACM is a progressive hereditary myocardial disease characterized by epicardial-to-transmural fibro-fatty replacement, progressive wall thinning, and a high risk of ventricular dysfunction, arrhythmias, and sudden cardiac death. The primary pathogenesis is driven by mutations in desmosomal genes (e.g., *PKP2*, *DSP*, *JUP*, *DSG2*, *DSC2*), which compromise cell adhesion and lead to myocyte detachment, as well as mutations in non-desmosomal genes (e.g., *DES*, *LMNA*, *TMEM43*, *TTNv*, *FLNC*) that disrupt cytoskeletal and nuclear envelope integrity. **Bottom left panel: Inflammatory Exacerbations (“Hot Phases”):** The clinical progression of ACM is frequently punctuated by episodic, acute inflammatory flares termed “hot phases.” These exacerbations are prominent in juvenile and pediatric populations, are frequently triggered by physical exercise (~50% of cases), and present with abundant inflammatory infiltrates that clinically overlap with acute myocarditis. Mutations in the *DSP* gene are the most common genetic substrate associated with these acute inflammatory presentations. **Central panel: The Inflammatory Cascade:** Myocyte necrosis and structural instability lead to the release of damage-associated molecular patterns (DAMPs), triggering a robust immune response. This cascade is orchestrated by macrophage activation, recruitment of mast cells, B cells, and T cells, and the proliferation of monocyte-derived macrophages. A complex pro-inflammatory signaling network—involving TNF-alpha, TGF-beta, interleukins, and MMPs operating through TLR, NF-kappa, MAPK, and Caspase-1 pathways—propagates inflammation to adjacent myocytes, fibroblasts, and endothelial cells, driving relentless cardiac remodeling and scarring. **Right panel: Clinical Relevance and Treatment:** The cycle of persistent inflammation is tightly linked to disease progression, although the exact prognostic value and strict causality remain active areas of investigation (guided by endomyocardial biopsy data). Crucially, the occurrence of a first “hot phase” episode represents a critical therapeutic window; timely administration of immunosuppressive treatment can significantly reduce the long-term risk of arrhythmias and heart failure, effectively mitigating the inflammatory impact to levels comparable to patients who have never experienced an acute exacerbation.

Nevertheless, the hot phases are supposed to impact the prognosis of patients with ACM but their role has yet to be demonstrated. A published observational cohort study by Gasperetti et al. [50] has evaluated the prognostic impact of inflammation through immunosuppressive treatment of the first hot-phase episode in 1014 patients with pathogenic or likely pathogenic DSP variants. Among them, 17.5% experienced ≥1 myocarditis-like episode and 35.6% received immunosuppressive therapy. Immunosuppressive treatment of the first episode was associated with a significantly reduced risk of ventricular arrhythmias and heart failure over a median follow-up of 6.4 years; this risk reduction was comparable to that of patients with no history of myocarditis-like episodes. Therefore, correctly treating the hot phase reduces the inflammation’s impact on prognosis and slows the progression of the inflammatory cardiomyopathy *[Figure 4]*.

## 6. Dilated Cardiomyopathy (DCM)

Dilated cardiomyopathy (DCM) is defined by the presence of left ventricular (LV) dilatation and systolic dysfunction unexplained solely by abnormal loading conditions (e.g., primary valve disease) or CAD [51]. The American Heart Association classifies DCM as genetic, mixed or acquired, whereas the European Society of Cardiology (ESC) groups cardiomyopathy into familial or nonfamilial forms [52,53]. Therefore, the etiology of DCM includes both genetic (primary DCM) and acquired factors (secondary DCM).

In primary non-inflammatory DCM, the most frequently implicated genes include LMNA, MYH7, TNNT2, TTN, RBM20, and BAG3 Truncating variants in TTN represent a common genetic cause of DCM, accounting for approximately 25% of familial cases and 18% of sporadic cases. Acquired causes encompass infections, toxic exposures, cancer therapies, endocrinopathies, pregnancy, tachyarrhythmias and immune-mediated diseases [54]. Despite growing insights into the disease, the role of inflammation and its contribution to disease pathogenesis pathways in DCM remain controversial. According to Padua Criteria [55], the differential diagnosis relies on myocardial tissue characterization using gadolinium-enhanced CMR. Key discriminative features include the extent and regional distribution of myocardial fibrosis assessed by late gadolinium enhancement (LGE), as well as the relationship between LGE burden and LV systolic dysfunction. Cipriani et al. [56] demonstrated that patients with DCM have higher LV volumes and mass, higher LVEF (46% versus 29%), and a greater amount of LV LGE (24.6% versus 13.1%) than DCM patients.

**Figure 5:**
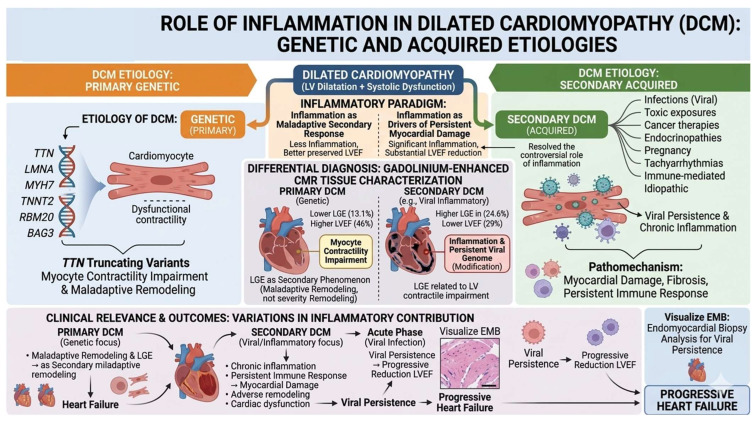
**The multi-faceted role of inflammation in primary (genetic) and secondary (acquired) dilated cardiomyopathy (DCM).** The schematic overview illustrates the distinct pathophysiological pathways, diagnostic features, and clinical outcomes differentiating primary and secondary etiologies of DCM. **Left panel (Genetic/Primary DCM):** Pathogenic variants in key sarcomeric and structural genes (e.g., *TTN* truncating variants, *LMNA*, *MYH7*, *TNNT2*, *RBM20*, *BAG3*) primarily disrupt cardiomyocyte contractility, leading to secondary maladaptive remodeling and heart failure, typically characterized by lower late gadolinium enhancement (LGE) and relatively higher left ventricular ejection fraction (LVEF). **Right panel (Secondary/Acquired DCM):** Extrinsic triggers—including viral infections, toxic exposures, chemotherapy, and immune-mediated processes—instigate chronic inflammation and persistent immune responses. This inflammatory cascade induces severe myocardial damage and adverse remodeling, leading to higher LGE and a more pronounced reduction in LVEF. **Central panel**: Cardiovascular magnetic resonance (CMR) tissue characterization via gadolinium enhancement serves as a pivotal differential diagnostic tool to distinguish primary contractility impairment from inflammation- and viral-persistence-driven tissue modification. Endomyocardial biopsy (EMB) remains the gold standard for visualizing viral persistence and inflammatory infiltrates in secondary DCM.

Therefore, patients with primary DCM typically exhibit less LV LGE, which represents a secondary phenomenon largely unrelated to LV contractile impairment. The LV dysfunction is primarily driven by impaired myocyte contractility, resulting from genetic mutations, whereas myocardial fibrosis reflects maladaptive remodeling rather than a determinant of disease severity [57] *[Figure 5]*.

## 7. Inflammatory Cardiomyopathy (CMi)

Inflammatory cardiomyopathy (CMi) is defined as myocarditis accompanied by cardiac dysfunction and ventricular remodeling. This condition is hallmarked by the infiltration of inflammatory cells into the myocardial tissue and exhibits a highly heterogeneous etiology. Although predominantly driven by viral infections, CMi can also be triggered by bacterial, protozoal, or fungal pathogens, as well as by a broad spectrum of toxic agents, pharmacological treatments, and systemic immune-mediated diseases [57].

Despite significant advances in clinical research, CMi continues to bear a poor prognosis when complicated by left ventricular dysfunction, overt heart failure, or severe arrhythmias. To date, the exact cellular and molecular mechanisms determining why certain patients achieve full recovery without residual myocardial injury, whereas others progress to end-stage dilated cardiomyopathy, remain a subject of intense investigation. Furthermore, the relative contributions of pathogen virulence, host genetic susceptibility, and environmental factors to disease progression are yet to be fully elucidated [58].

**Figure 6:**
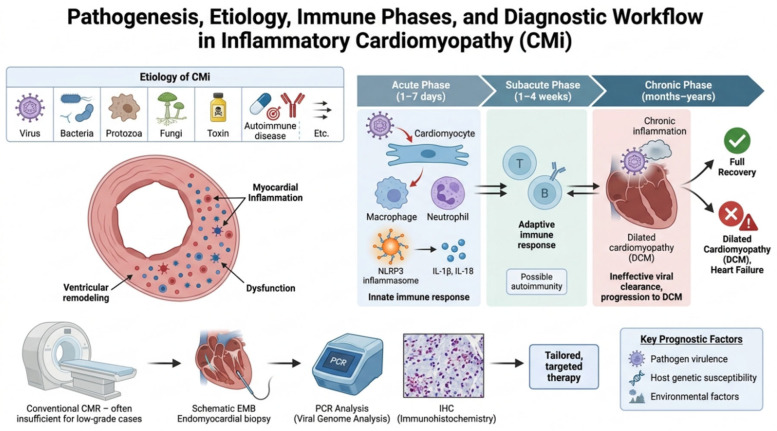
**Etiology, immune phases, and diagnostic workflow in inflammatory cardiomyopathy (CMi).** CMi is triggered by diverse etiologies (infectious, toxic, autoimmune), causing myocardial inflammation and ventricular remodeling. Pathogenesis progresses from an acute phase (innate immunity, NLRP3 inflammasome activation, IL-1β/IL-18 release) through a subacute phase (adaptive T/B-cell response) to a chronic phase (persistent inflammation leading to dilated cardiomyopathy [DCM] or recovery). Clinically, when conventional CMR is insufficient, endomyocardial biopsy (EMB) with PCR and immunohistochemistry (IHC) guides tailored therapy. Outcomes depend on pathogen virulence, host genetics, and environmental factors.

From a clinical perspective, achieving an accurate diagnosis poses a considerable challenge. Persistent, low-grade myocardial inflammation can be elusive when relying solely on conventional non-invasive imaging modalities, such as cardiovascular magnetic resonance (CMR). Consequently, endomyocardial biopsy (EMB), coupled with quantitative viral genome analysis and immunohistochemistry, remains a crucial diagnostic tool for establishing the underlying etiology of CMi at the cellular level and tailoring targeted therapeutic interventions *[Figure 6]*.

While in non-inflammatory DCM, immune dysregulation may act as significant modifier of disease severity and progression, in CMi inflammation is the primary trigger of the disease [59].

Persistent subclinical inflammation resulting from chronic viral infection has been implicated in the progression of acute myocarditis to DCM [60]. Kuhl et al. [61] analyzed EMB samples from 245 patients with DCM and detected viral genomes from EMBs of 165 (67.4%). Subsequently, viral genome persistence was associated with a progressive reduction in LVEF [62], but the causal role of these viral genomes on the impairment of systolic function and clinical outcome remains controversial. In addition to the role of the viral genome, acute myocarditis is driven by an abnormal immune response involving both innate and adaptive immunity. The innate immune system provides the initial, non-specific defense through pattern-recognition receptors and inflammasomes—particularly NLRP3—which amplify inflammation via cytokines such as IL-1β and IL-18. The adaptive immune system, mediated by T and B cells, mounts a pathogen-specific response but may also contribute to autoimmune myocardial injury. Dysregulation or persistence of these immune responses can result in excessive inflammation, myocardial damage, adverse remodelling, and cardiac dysfunction [63]. The pathogenic process of viral inflammatory cardiomyopathy can be conceptually divided into three phases. The acute phase of viral entry into the cell and activation of the innate immune response lasts approximately 1–7 days; the subacute phase is characterized by the adaptive immune response, spanning 1–4 weeks. The chronic phase persists from months to years, and it is correlated with delayed or ineffective viral clearance combined with chronic inflammation leading to DCM [64].

## 8. Role of Endomiocardial Biopsy (EMB)

While CMR has emerged as a cornerstone in the non-invasive diagnostic workup of myocardial inflammation, it presents notable pitfalls, particularly in low-grade or highly focal inflammatory processes. Consequently, endomyocardial biopsy (EMB) retains a critical and irreplaceable role in the diagnostic algorithm. The diagnostic sensitivity of CMR is highly variable and heavily dependent on the clinical presentation and the underlying cellular mechanisms of tissue injury. Specifically, while CMR exhibits high sensitivity in identifying acute, infarct-like myocarditis characterized by extensive cell necrosis and prominent interstitial edema, its diagnostic accuracy drops significantly in cardiomyopathic and arrhythmic presentations.

In these latter scenarios, the inflammatory process is often limited in spatial extent and disease activity. Furthermore, when myocardial injury is primarily driven by apoptosis rather than necrosis, cell membrane integrity is preserved, which inherently limits the expansion of the interstitial space and substantially reduces CMR signal abnormalities. As a result, the relatively low contrast and spatial resolution of standard CMR techniques can yield false-negative results in low-grade inflammatory conditions [65]. Therefore, whenever there is a strong clinical suspicion of severe or progressive disease—such as unexplained electrical instability or rapid hemodynamic deterioration—negative CMR findings do not preclude the presence of active inflammation, underscoring the necessity of EMB for a definitive histological, immunohistochemical, and molecular diagnosis.

Although the non-invasive diagnosis of myocardial diseases has advanced significantly through state-of-the-art imaging techniques, endomyocardial biopsy (EMB) remains an irreplaceable diagnostic tool for specific clinical phenotypes. Within the spectrum of cardiomyopathies, recent ESC guidelines emphasize that EMB is not indicated as a routine screening procedure for all patients. However, its application is strongly supported and recommended in clinical scenarios characterized by a well-founded suspicion of a disease for which targeted, disease-modifying therapies can be initiated [52]. In this context, the histological, immunohistochemical, and molecular analysis of myocardial tissue is paramount to establishing a definitive diagnosis in conditions such as cardiac amyloidosis, Anderson–Fabry disease, hemochromatosis, or inflammatory cardiomyopathies, thereby ensuring timely and tailored therapeutic interventions.

In the specific setting of myocarditis, EMB represents the current diagnostic gold standard, bridging the gap between non-specific inflammation and a defined molecular etiology. According to the 2025 ESC guidelines, the procedure is strongly indicated in patients presenting with rapid hemodynamic deterioration, including acute heart failure, cardiogenic shock, or life-threatening ventricular arrhythmias. In these high-risk scenarios, EMB is crucial for either excluding or confirming severe phenotypes that mandate immediate intervention—such as giant cell myocarditis or eosinophilic myocarditis—both of which require prompt and aggressive immunosuppressive therapy [66].

Endomyocardial biopsy (EMB) currently retains a highly selected but clinically relevant role in the diagnostic work-up of infiltrative and storage cardiomyopathies, particularly when non-invasive algorithms are not applicable, inconclusive, or discordant.

In cardiac amyloidosis, EMB should be considered in patients with a high clinical suspicion of disease when a monoclonal protein is detected, as demonstrated by an abnormal serum free light-chain ratio and/or positive serum or urine immunofixation, since the non-biopsy diagnostic pathway for transthyretin amyloid cardiomyopathy is no longer valid in this setting and tissue confirmation with amyloid typing becomes mandatory. Similarly, EMB is indicated when bone-tracer scintigraphy is negative, equivocal, unavailable, or discordant with clinical, laboratory, or imaging findings, particularly when extracardiac biopsy is negative or non-diagnostic despite persistent suspicion of cardiac involvement. In this context, EMB remains the diagnostic gold standard for cardiac amyloid deposition, with very high diagnostic accuracy when adequate sampling is performed. Histologically, amyloidosis is defined by extracellular Congo red-positive deposits showing apple-green birefringence under polarized light, but the identification of the amyloid precursor is essential for therapeutic decision-making. Therefore, biopsy samples should undergo amyloid subtyping using immunohistochemistry or immunofluorescence in experienced laboratories, with liquid chromatography–tandem mass spectrometry regarded as the preferred reference method, particularly when immunostaining is inconclusive or discordant [67,68].

EMB may also be useful in selected cases of suspected Anderson–Fabry disease, especially when the diagnosis remains uncertain despite suggestive clinical and imaging features, such as in heterozygous females with normal or borderline α-galactosidase A activity, or in patients carrying a GLA variant of uncertain significance in whom histological confirmation would influence the initiation of disease-specific therapy. In Fabry cardiomyopathy, light microscopy may show cardiomyocyte vacuolization due to lysosomal glycosphingolipid storage, whereas electron microscopy provides a key diagnostic contribution by demonstrating characteristic intralysosomal globotriaosylceramide deposits, and should therefore be requested when storage or metabolic cardiomyopathy is suspected. From a practical standpoint, diagnostic yield is strongly influenced by sampling adequacy; at least three, and preferably four to six, myocardial fragments should be obtained because disease involvement may be patchy and insufficient sampling remains a major cause of false-negative results. Although EMB is an invasive procedure, its risk is low in experienced centers, with major complication rates generally reported below 1%, supporting its use when the expected diagnostic and therapeutic impact justify the procedure [19,20,21,22,23,24,25,69].

Furthermore, the clinical utility of EMB extends beyond mere morphological evaluation. The application of immunohistochemical techniques to quantify leukocyte infiltrates (e.g., HLA antigen expression) and the use of polymerase chain reaction (PCR) assays for the detection of viral genomes are essential steps in characterizing the inflammatory microenvironment. This molecular differentiation is a mandatory prerequisite for distinguishing between an active viral infection and a chronic immune-mediated inflammatory response, ultimately guiding the clinician through the complex transition toward etiology-specific treatments, such as antiviral agents or personalized immunosuppressive regimens.

## 9. Conclusions

The traditional paradigm of cardiomyopathies as exclusively structural or metabolic disorders is evolving. Current evidence underscores that chronic, low-grade myocardial inflammation is an active pathophysiological driver across hypertrophic cardiomyopathy, Anderson–Fabry disease, cardiac amyloidosis, arrhythmogenic cardiomyopathy, and dilated cardiomyopathy. Endogenous triggers, including metabolic storage products, amyloid fibrils, mechanical stress, and viral genomes, act as damage-associated molecular patterns. These signals activate innate immune cascades, including Toll-like receptors, the NF-κB pathway, and the NLRP3 inflammasome. This cascade establishes a pro-inflammatory microenvironment that promotes fibroblast reprogramming, extracellular matrix deposition, and progressive maladaptive remodeling.

Recognizing inflammation as a core mechanism provides a crucial framework for improved risk stratification and highlights the potential for targeted immunomodulatory therapies to alter disease trajectories. Future studies are needed to clarify whether inflammatory pathways play a causal role in restrictive and non-dilated cardiomyopathy, or represent secondary phenomena related to disease stage.

## Data Availability

No new data were created or analyzed in this study.
